# Assessment of Premature Failures in Concrete Railway Ties: A Case Study from Brazil

**DOI:** 10.3390/ma18132994

**Published:** 2025-06-24

**Authors:** Eliane Betânia Carvalho Costa, Maria Eduarda Guedes Coutinho, Rondinele Alberto Dos Reis Ferreira, Antonio Carlos Dos Santos, Luciano Oliveira

**Affiliations:** 1 Faculty of Civil Engineering, University Federal of Uberlandia, Uberlandia 38408-100, MG, Brazil; maria.coutinho@ufu.br (M.E.G.C.); rondinelealberto@gmail.com (R.A.D.R.F.); acds@ufu.br (A.C.D.S.); 2 VALE S.A, Vitória 22250-145, ES, Brazil; oliveira.luciano@vale.com

**Keywords:** sleepers, internal swelling reaction, ettringite, alkali–silica reaction, concrete

## Abstract

Prestressed concrete railroad ties are the global standard for railway infrastructure due to their structural stability, durability, and cost-effective maintenance. However, their long-term performance is often compromised by premature deterioration. This study investigates the degradation of prestressed concrete railways ties from a Brazilian rail line after ten years of natural exposure, emphasizing critical implications for infrastructure maintenance. Two groups of ties, separated by 30 km, were analyzed through physical property assessments, petrography, X-ray diffraction (XRD), and scanning electron microscopy/energy dispersive spectroscopy (SEM/EDS). The results reveal that deterioration was driven by the combined effects of alkali–silica reaction (ASR) and sulfate attack, confirmed by the presence of (N, C)ASH gels, ettringite crystallization, and cryptocrystalline materials within cracks and voids. Prestressing-induced stresses and environmental moisture further accelerated degradation, leading to a 66% reduction in mechanical strength in the T1 group. These findings demonstrate that internal swelling reactions and moisture exposure synergistically accelerate deterioration in prestressed concrete ties, particularly in low-prestress, poorly drained zones.

## 1. Introduction

Railroad ties are fundamental structural elements in railway infrastructure, performing critical functions including rail alignment maintenance, dynamic load accommodation, and force distribution to the ballast. The global railway tie market, valued at USD 806 million in 2023, is projected to grow to USD 1064 million by 2030, representing a compound annual growth rate (CAGR) of 4% [1].

Railroad ties are primarily manufactured using timber, concrete, or, less commonly, steel [1,2]. Among these, prestressed concrete ties have emerged as the global standard, due to their combined structural and economic advantages, including enhanced stability, load-bearing capacity, durability, and lower lifecycle costs compared to alternatives [3,4,5]. However, these benefits are accompanied by significant challenges. Research indicates that 2–5% of concrete ties require replacement annually due to premature failure [6,7]. In Indian railways, visible cracking develops within 6–9 years of manufacture, even in unused ties stored adjacent to tracks [8]. Brazilian railway operators report similar deterioration patterns, with over 1 million tie replacements conducted during the past decade, with approximately 60% involving premature aging of prestressed concrete ties [9].

A notable incident highlighting concrete tie failures occurred during the 2022 Garmisch-Partenkirchen train derailment [7]. Investigators from the Federal Bureau for Railway Accident Investigation attributed the accident to compromised prestressed concrete ties, which experienced tension loss and structural failure [7,10,11]. Subsequent analysis revealed that the aggregate type used in the concrete may have contributed to the failures, prompting Deutsche Bahn to expand its investigation beyond manufacturing defects to evaluate material compatibility issues [10,11]. As a preventive measure, Deutsche Bahn initiated the replacement of approximately 44,000 concrete ties, many only at mid-lifecycle [12].

According to Ferdous and Manalo, the most common types of failures in concrete ties worldwide are rail-seat deterioration (RSD) and longitudinal cracking [13]. These defects mainly appear near rail seats and at the center of ties [14], driven by key factors such as ballast particle abrasion, hydraulic pressure fluctuations, and freeze–thaw cycles [8]. Synergistic deterioration mechanisms, particularly delayed ettringite formation (DEF) coupled with alkali–silica reaction (ASR), have been extensively documented as causes of cracking [8,15,16,17].

Microstructural analyses from international studies demonstrate the critical role played by internal swelling reactions in premature prestressed concrete tie deterioration. Swedish ties produced between 1992 and 1996 exhibited microcracks and interfacial gaps consistent with delayed ettringite formation (DEF) [18]. In Indian railways, SEM revealed ettringite deposition at aggregate–paste interfaces, resembling ASR gel patterns [8]. On the Nanjing–Shanghai line in China, ASR was identified as the cause of map and longitudinal cracking in prestressed ties [15]. Similarly, a combination of ASR/DEF damage was found in 15-year-old ties used in the Korean high-speed rail network [17]. Environmental exposure—especially moisture ingress and wetting–drying cycles—exacerbates ASR and sulfate attack [19,20,21]. Crack networks facilitate the penetration of aggressive agents, with damage severity governed by crack morphology [22].

Building upon these international findings, this study systematically investigates the deterioration mechanisms affecting prestressed concrete ties along a northern Brazilian railway after a decade in service. By employing a multi-technique characterization approach, we provide a comprehensive diagnosis aimed at enhancing durability, reducing long-term maintenance costs, and generating fundamental data for the predictive modeling of concrete ties’ behavior under tropical conditions. The experimental results and degradation patterns identified offer valuable input for developing more accurate service life models and finite element simulations of tie performance. Improving the service life of concrete ties not only optimizes infrastructure performance but also mitigates environmental impacts—including natural resources’ depletion, solid waste generation, and carbon dioxide (CO_2_) emissions—thus supporting broader sustainability goals in railway engineering.

## 2. Experimental Program

This study examined prestressed concrete ties measuring 2800 mm in length, manufactured in 2011 using the long-line production method. The specimens were retrieved after approximately ten years of service (2021) from a railway line in northern Brazil (6°04′14″ S, 49°54′15″ W) at an elevation of 156 m above sea level. The study area features a tropical climate with a mean annual temperature of 26.1 °C, relative humidity range of 53–88%, and annual precipitation of 1564 mm.

**Figure 1:**
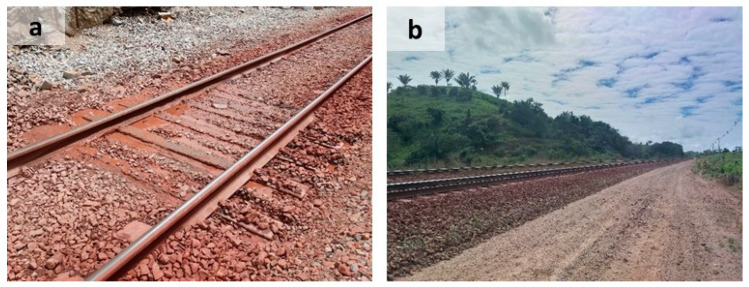
Location where concrete tie groups were installed on the railway: (**a**) T1; (**b**) T2.

While both tie groups (T1 and T2) experience identical traffic loads and regional climate conditions, their microenvironments differ significantly in terms of drainage characteristics. As shown in Figure 1, T2 ties are positioned on an elevated track segment with efficient lateral runoff, whereas T1 ties are situated in a depression exhibiting chronic water accumulation due to inadequate drainage. These distinct hydrological conditions create substantially different saturation regimes that directly correlate with the observed differential degradation patterns.

The concrete ties were manufactured by the same company using similar materials and production conditions, though from different batch production. The ties were produced with CPV-ARI Portland cement (95–100% clinker and calcium sulfates, with 5% limestone filler), according to the Brazilian standard [23]. Fine and coarse aggregates were sourced from a deposit in central-western Brazil: the fine aggregate consisted of washed river sand (4.8–0.15 mm particle size) with a specific gravity of 2.54 g/cm^3^ and a density of 1430 kg/m^3^, while the coarse aggregate comprised crushed granite gravel (19 mm maximum size) with a specific gravity of 2.57 g/cm^3^, a density of 1400 kg/m^3^, and a water absorption rate of 1%. The concrete mixture also included ADVA^®^ 518, a type 2 water reducer with setting- and strength-accelerating properties. Key characteristics of the concrete mix design are summarized in Table 1, based on information obtained from the manufacturer’s internal databooks provided for this investigation.

**Figure 2:**
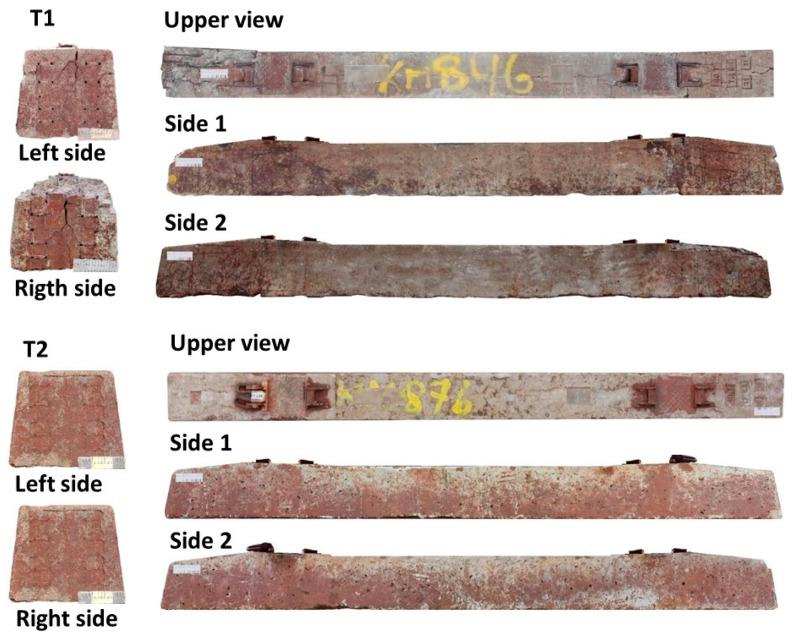
Evaluated surfaces of concrete tie specimens (T1 and T2).

Ten concrete ties from each group were selected from the railway track based on their prevalence of surface cracks observed during preliminary inspection. A substantial amount of surface material was present on the selected ties; this was removed via plastic brushing and water rinsing to obtain a clean surface for analysis. Figure 2 illustrates the evaluated faces of the concrete ties for each group.

**Figure 3:**
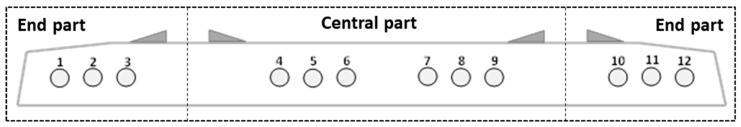
Core extraction pattern showing locations of 50 mm × 100 mm cores in tie specimens (six from central region, three from each end). The core numbering follows this sequence: three cores (1–3) at one end, six cores (4–9) in the central region, and three cores (10–12) at the opposite end of the concrete tie.

Concrete cores (50 mm diameter × 100 mm length) were extracted following ABNT NBR 7680 [24] using a diamond coring-drilling machine. From each element, six cores were taken from the central region, with three additional cores collected from each end (Figure 3). All drilling operations were performed perpendicular to the original concrete casting direction. To preserve sample quality, a minimum center-to-center spacing of 10 mm was maintained between adjacent extraction points, reducing stress-induced microcracking and avoiding contact with reinforcement steel.

The experimental program was designed to comprehensively evaluate both the causes and effects of internal concrete deterioration through three complementary approaches. Mechanical testing assessed residual performance by quantifying strength loss and structural degradation in ties after prolonged service exposure. Chemical analysis focused on detecting deleterious compounds, including chlorides (which promote steel corrosion), sulfates (responsible for expansive reactions), and soluble salts (associated with efflorescence). Transport property measurements evaluated durability loss by tracking water absorption and chloride migration rates, which revealed how internal cracking from swelling reactions increases concrete permeability. This enhanced permeability creates a damaging cycle by accelerating the ingress of harmful agents and expanding microcrack networks. Together, these methods provide a complete understanding of current tie condition while predicting long-term degradation behavior.

### 2.1. Preliminary Inspection

**Figure 4:**
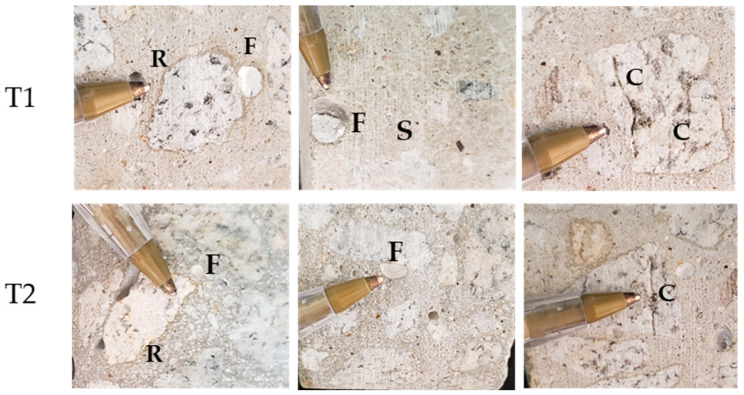
Macroscopic distress features in T1 and T2 cores with scale reference (1 cm pen type shown). All features illustrated are associated with internal swelling reactions: (C) ASR/sulfate-induced cracking; (F) calcium carbonate efflorescence; (R) ASR rims around aggregates; and (S) iron oxide staining in mortar from pyrite oxidation.

Preliminary macro-examination of the extracted cores revealed several characteristic features in both T1 and T2 specimens (Figure 4). Cracking was observed in both aggregates and mortars, accompanied by distinct reaction edges surrounding aggregates and white deposits filling pore spaces. While these features were consistently present in T1 specimens, visual analysis detected no reaction edges on coarse aggregates in T2 specimens. Notably, neither specimen type showed evidence of corrosion in the embedded steel reinforcement. These observations informed us of the subsequent selection of samples for detailed testing.

### 2.2. Mechanical Tests

Compressive strength was determined using a 600 kN universal testing machine following ABNT NBR 5739 [25], with a controlled stress rate of 0.45 MPa/s. Six cylindrical specimens (50 × 100 mm) were tested from each concrete tie: three from the central region (positions 4–9, Figure 2) and three from one end. Specimens were extracted from the core’s central portion to ensure representativeness.

The dynamic elastic modulus was evaluated via ultrasonic wave propagation [26] using a 54 kHz piezoelectric transducer (contact area: 16.62 cm^2^). The same specimen geometry and sampling locations were maintained for consistency with compressive testing.

### 2.3. Determination of Soluble Salts, Sulfates, and Chlorides

Following mechanical testing, concrete samples were collected from both central and end regions of each tie (four replicates per element) for chemical analysis. Aqueous extracts were prepared using distilled water in a 1:5 solid-to-liquid ratio, followed by quantitative analysis via (i) argentometric titration with 0.1 M AgNO_3_ for chloride content and (ii) turbidimetric spectrophotometry (Pharo 300 Spectroquant^®^, Darmstadt, Germany) with BaCl_2_ precipitation for sulfate quantification [27]. This systematic approach enabled the comprehensive evaluation of soluble salt distribution while accounting for potential spatial variations within the ties.

### 2.4. Water and Chloride Transport Properties

Capillary water absorption was measured on 50 mm diameter cylindrical specimens following EN ISO 15148 [28]. The lateral surfaces were sealed with silicone up to 30 mm height, exposing one circular face to water under a constant 5 mm head. Mass changes were recorded at intervals from 1 min to 72 h (1, 3, 5, 7, 10, 15, 30, 60, 90, 120, 240, 1440, 2880, and 4320 min). The water absorption coefficient (C_90_) was determined from the initial slope of the absorption curve (kg·m^−2^ vs. √time in h^1/2^), representing the capillary uptake during the first 90 min.

The chloride migration coefficient (D_nssm_) was assessed via NT BUILD 492 [29] using 50 mm core specimens. After Ca(OH)_2_ vacuum saturation, the samples underwent electrolytic testing (0.3 N NaOH/10% NaCl) with the voltage adjusted from 30 V based on the current response. Post-test AgNO_3_ spraying revealed penetration depth, enabling D_nssm_ calculation incorporating voltage, thickness, duration, and temperature. This adaptive method yields reliable transport data for low-permeability concretes. The resulting migration coefficients serve as reliable input parameters for durability modeling and service life predictions.

### 2.5. Microstructural Analysis

Petrographic analysis was performed on 50 mm diameter core samples (from both end and central regions) following ASTM C856M-20 [30] and ABNT NBR 15577-3 [31]. Initial macroscopic examination using a binocular microscope (up to ×200 magnification) identified cracking patterns, void distribution, and discoloration, guiding thin-section selection. Subsequent microscopic analysis employed transmitted light (plane- and cross-polarized) and fluorescent illumination (up to ×630) to characterize aggregate composition (including reactivity potential and carbonate content), microstructural features (crack networks, gel formation, and void systems), and cement paste integrity (focusing on alteration products and interfacial transition zones). This multiscale approach enabled the comprehensive assessment of material degradation mechanisms.

X-ray diffraction (XRD) analysis was performed on crushed specimens from mechanical testing to characterize hydration products and degradation phases in the cementitious matrix. Representative samples from central and end regions of each concrete tie were prepared through sequential particle size reduction: primary grinding (HERZOG oscillating disk mill), sieving (<75 μm), and final pulverization (<5 μm). XRD measurements employed a Shimadzu LabX XRD-6100 diffractometer (Kyoto, Japan) with CuKα radiation (40 kV, 30 mA), scanning 4–70° 2θ at 0.02° increments (0.6 s/step). Phase identification occurred via the ICSD database.

Microstructural characterization was completed using scanning electron microscopy with energy-dispersive X-ray spectroscopy (SEM-EDS). Sample preparation involved sectioning with a water-cooled diamond saw, immediate rinsing with deionized water, drying at 50 °C to constant mass (±0.1 g stability over 24 h), and sputter-coating with 15 nm and gold-coated. Analysis was performed using a ZEISS EVO MA10 SEM operated at 20 kV (Oberkochen, Germany) and Oxford Instruments X-Max 51-ADD0048 EDS detector (Abingdon, UK), with six representative regions examined per concrete tie. EDS analysis was also conducted to identify the main elemental composition.

## 3. Results and Discussion

### 3.1. Mechanical Properties

The experimental results revealed significant differences in the mechanical properties between the two concrete ties after 10 years in service (Table 2). The T1 specimens exhibited the lowest mechanical performance, with notably reduced strength compared to T2. In contrast, the T2 specimens displayed a 3% higher compressive strength in their central regions but suffered a substantial 40% decrease at the end.

Although T1 and T2 demonstrated similar 28-day compressive strength, their long-term performance diverged significantly due to environmental conditions, particularly moisture availability, which played a critical role in accelerating the deterioration mechanisms. In T1, poor drainage led to sustained moisture exposure, exacerbating internal swelling reactions (e.g., alkali–silica reaction or sulfate attack). This persistent moisture ingress not only increased expansive pressures but also promoted progressive chemical and microstructural damage. Over time, these effects manifested as crack propagation, increased porosity, and interfacial bond weakening, collectively contributing to strength loss.

Conversely, T2’s well-drained environment restricted moisture ingress, slowing down reaction kinetics and preserving the concrete’s microstructure. The limited water availability reduced expansive pressure and prevented cumulative damage, enabling T2 to retain higher strength. Thus, despite identical initial properties, the disparity in long-term performance stems from differences in the deterioration rates driven by site-specific moisture conditions.

**Figure 5:**
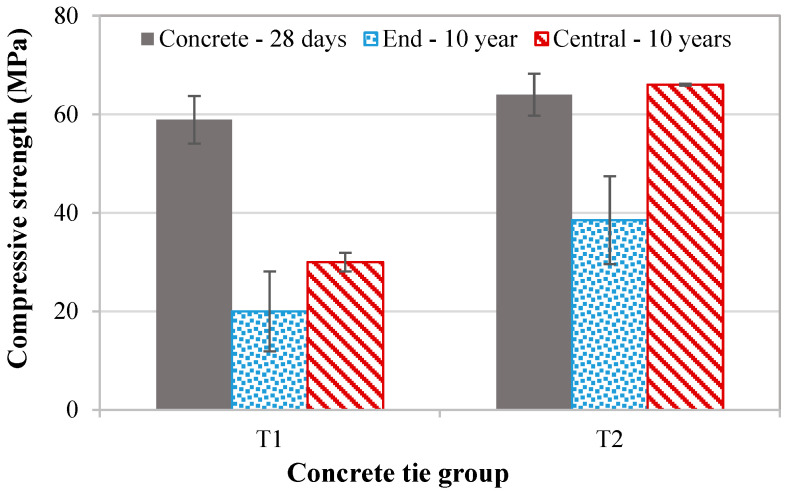
Comparison of 28-day compressive strength versus in-service performance after 10 years for concrete ties T1 and T2: laboratory-cured control specimens, core samples from central tie regions, and core samples from end regions. Error bars represent standard deviation.

Spatial performance variation was also evident within each tie, with the end regions consistently underperforming compared to the central sections (Figure 5). This behavior can be attributed to the non-uniform prestress distribution inherent to prestressed concrete ties. The central region, subjected to higher prestress levels, develops to counterbalance the maximum structural stresses during service. The prestress tension progressively decreases toward the tie ends, creating a pronounced mechanical gradient along the tie length. This prestress distribution pattern critically influences structural integrity. The reduced prestress at the ends reduces crack resistance, leading to microcrack formation, as documented in [32].

### 3.2. Concentration of Soluble Salts, Sulfates, and Chlorides

Table 3 shows variations in soluble salt content (0.96% to 2.17%) among the concrete tie specimens. T1 exhibited consistently higher salt concentrations than T2 across all sampling locations. Notably, T2 displayed significant spatial variability, with end-region samples containing 1.8–2.1 times more soluble salts than central sections, suggesting differential transport mechanisms along the tie length.

Sulfate levels followed similar trends (T1: 0.10%; T2: 0.05–0.11%), while chlorides remained below detection limits (<0.0062%) in all regions. These salts may originate from the cementitious matrix, absorbed water, atmospheric pollutants (particularly sulfates), or microbial activity [33]. Crystallization pressures from supersaturated solutions—driven by capillary action, evaporation, or wetting–drying cycles [34,35]—can induce concrete damage [18], with deterioration severity depending on salt chemistry, transport kinetics, and the concrete’s pore structure characteristics [35].

T1 ties had a significantly higher soluble salts concentration (≈2.18%) than T2 (0.96–1.26%), particularly in the central region. Similarly, the sulfate contents were also elevated in T1. These differences align with the visual observations of more extensive cracking in T1 (see Figure 2), especially at the tie ends, where the carbonation effects are typically stronger. Though chloride contents were undetectable in all regions, the presence of soluble sulfates—combined with high moisture exposure—likely contributed to crystallization-induced damage. Though absolute differences are small, sulfates can induce ettringite/thaumasite formation in moist conditions, potentially contributing to T1’s worse cracking. Sulfates chemically react with cement hydrates, causing expansion and cracking. Their effect is secondary to soluble salts here but may synergistically worsen damage in T1.

The higher salt concentration in T1, paired with its higher permeability, facilitated the ingress of aggressive agents, creating a self-accelerating deterioration cycle through multiple pathways: ASR acceleration from increased alkali availability; sulfate attack leading to ettringite/thaumasite formation; and reinforcement corrosion risks despite low chloride levels.

### 3.3. Evaluation of Water and Chloride Transport Properties

**Figure 6:**
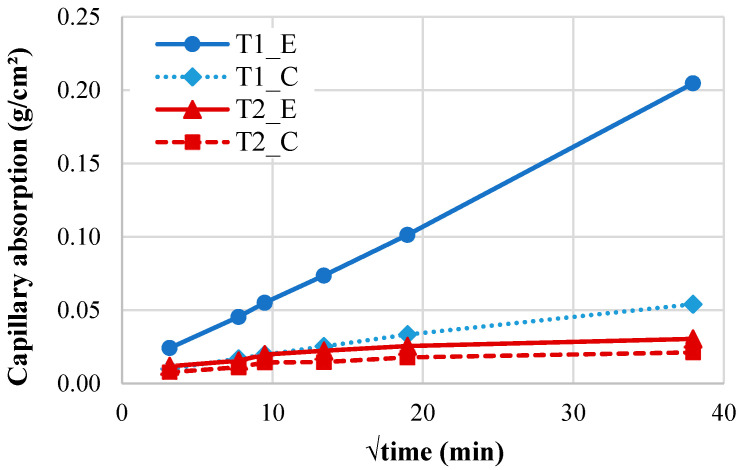
Capillary water absorption versus square root of time for specimens extracted from concrete ties (Tn_E: end region; Tn_C: center region), where n denotes the tie group number (T1 or T2).

Figure 6 presents the capillary water absorption behavior of the concrete ties plotted against the square root of time. The results demonstrate significantly lower sorptivity in T2 compared to T1 (*p* < 0.05). Notably, specimens extracted from the tie ends exhibited markedly higher absorption coefficients than central sections, with T1_E (end-region specimen) showing the most pronounced capillary uptake.

**Figure 7:**
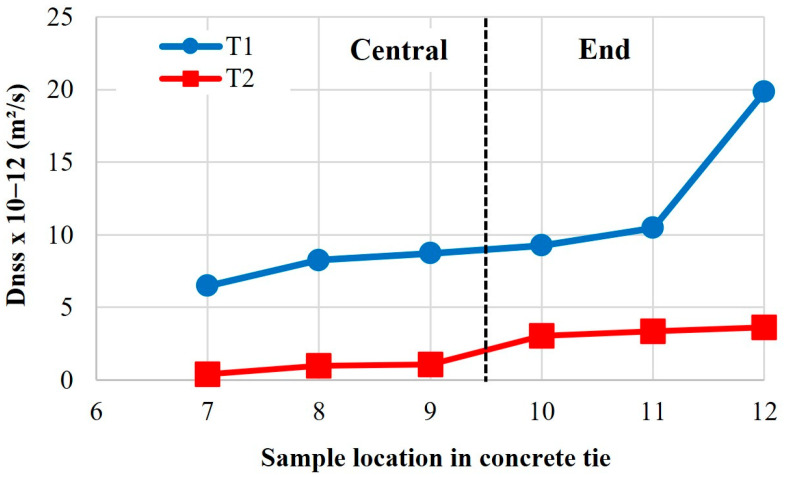
Chloride migration coefficient of specimens extracted from concrete ties: central region: 7; 8; 9 and end region: 10; 11; 12.

**Figure 8:**
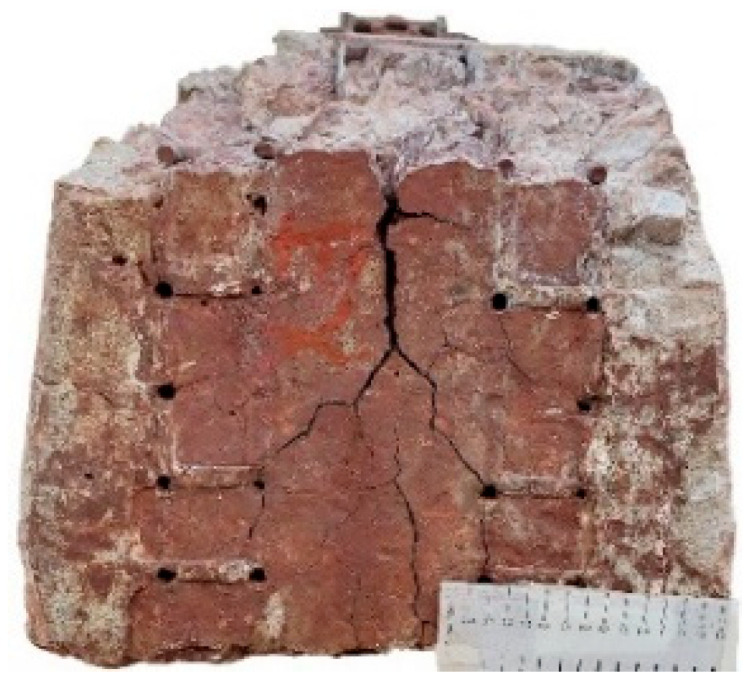
Severe deterioration patterns at T1 tie ends, including localized defects in sample 12.

Figure 7 shows the chloride migration of concrete ties for different sample locations after 10 years in service. The data reveal clear differences in the chloride migration coefficients (D_nss_) between central and end-region samples from two concrete ties (T1 and T2). In both cases, end-region samples (10–12) exhibited significantly higher D_nss_ values than central samples (7–9), suggesting greater chloride penetration susceptibility at the tie ends. Notably, T1 showed much higher overall coefficients (6.5–19.85 × 10^−12^ m^2^/s) compared to T2 (0.42–3.62 × 10^−12^ m^2^/s), indicating potential differences in degradation mechanisms. A pronounced outlier was observed in T1_end sample 12 (19.85 × 10^−12^ m^2^/s), which nearly doubled the next highest value, attributable to localized defects from severe deterioration, as visually confirmed in Figure 8. In contrast, T2 exhibited lower and more uniform values, reflecting its superior chloride resistance.

The performance difference between T1 and T2 ties originates from fundamental variations in microenvironmental exposure and resultant deterioration mechanisms. T1 exhibited significantly greater capillary absorption (Figure 6) and chloride migration than T2 (Figure 7), directly attributable to its pronounced microcracking networking, especially in the end regions where crack density exceeded central zones (Figure 8), corresponding to 2.1x higher fluid transport rates. The microstructural analysis (detailed in subsequent sections) reveals that T1’s degradation primarily resulted from synergistic moisture-driven ASR and sulfate attack, generating expansive phases that preferentially damaged the end regions under combined environmental and mechanical stresses. In contrast, T2’s microstructure demonstrates effective resistance to these deterioration pathways, maintaining durability through restricted crack development and fluid penetration. These findings suggest that microenvironmental exposure significantly influences transport behavior through its effects on microstructural deterioration.

### 3.4. Petrographical Examination

**Figure 9:**
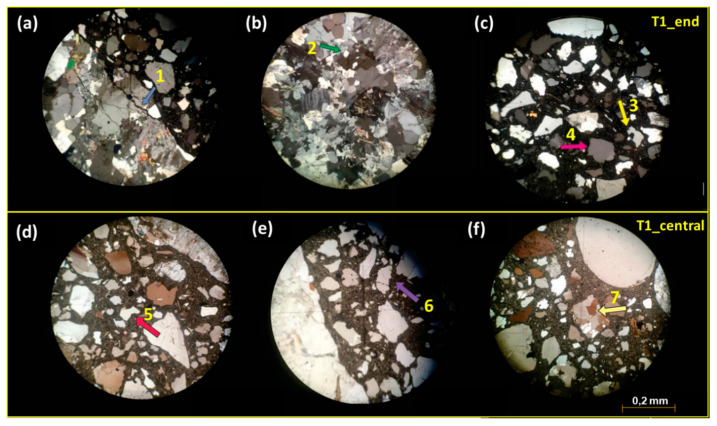
Thin section photomicrographs of T1 concrete tie samples (end and center regions): (**a**) crack in coarse aggregate, filled with cement paste and cryptocrystalline material (1—blue arrow); (**b**) polycrystalline quartz aggregate showing undulating extinction and slightly orientation (2—green arrow); (**c**) quartz aggregate with corroded edges (3—yellow arrow) and adjacent crystals exhibiting undulose extinction (4—pink arrow); (**d**) subangular quartz fine aggregate displaying corrosion and reaction rims (5—pink arrow); (**e**) microfractured quartz coarse aggregate containing cement inclusions (6—lilac arrow); (**f**) polycrystalline quartz fine aggregate (7—yellow arrow) with early-stage carbonatation in cement paste.

**Figure 10:**
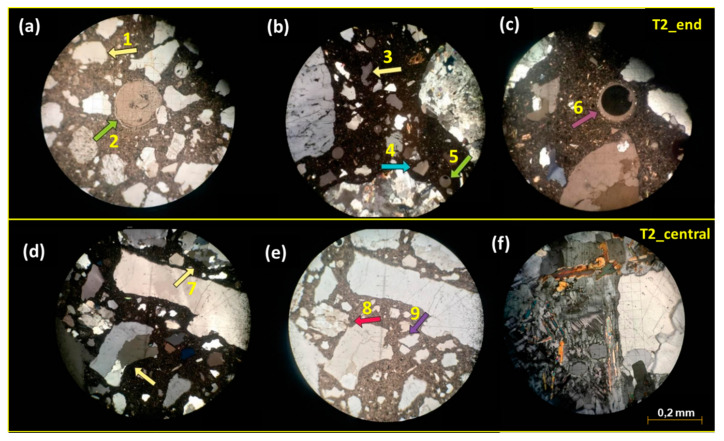
Thin section photomicrographs of T2 concrete ties samples (end and center regions): (**a**) quartz fine aggregates with corroded/altered edges (1—yellow arrow), voids filled by cryptocrystalline products, and a thin film of gel around the aggregate (2—green arrow); (**b**) subangular quartz with corrosion features and reaction rim (3—yellow arrow), undulose extinction (4—blue arrow), and voids-filling gel and cryptocrystalline material (5—green arrow); (**c**) gel-lined voids with edge alteration and early-stage carbonation (6—lilac arrow); (**d**) monocrystalline and polycrystalline quartz coarse aggregate with undulose extinction (7—yellow arrows); (**e**) microfractured quartz fine aggregate with corrosion rims (8—pink arrow), and edge gel films (9—lilac arrow); (**f**) coarse aggregate assemblage of altered plagioclase, augite, and quarts showing lobate contact.

Table 4 summarizes the main petrographic characteristics of the concrete ties, with the supporting microscopic images of T1 and T2 samples shown in Figure 9 and Figure 10. The concrete ties exhibit good density and compactness without signs of disaggregation, displaying a uniform gray-to-light-gray coloration. Both groups share similar compositional characteristics.

Macroscopic examination reveals that all aggregate particles are completely embedded in cementitious matrix, exhibiting strong interfacial bonding with no observable detachment. Coarse aggregates primarily consist of granite, with T2 samples containing both granite and granitoid. Deformation regions featuring fine-textured crystals suggest a single source rock, likely from a quarry that experienced deformation and hydrothermal alteration processes [36]. The mineralogical composition includes quartz, plagioclase, alkali feldspar, micas, and subordinate amphiboles (e.g., biotite). Medium monocrystalline quartz crystals frequently display undulose extinction and lobate contacts (Figure 9b,c and Figure 10b,d), indicating recrystallization due to changes in temperature, pressure, or chemical environment [37].

Fine aggregates predominantly consist of monocrystalline and polycrystalline quartz showing undulose extinction and fracturing, with sinuous and corroded edges observed in both groups (Figure 9d and Figure 10a). Subordinate minerals include pyroxene, microcline, elongated plagioclase, and phyllosilicates (muscovite, biotite, and phlogopite). While T1_central and both T2 samples contain lithic material and filled bubbles, some samples exhibit intra- and intercrystallyne microcracks partially filled with cement paste. Although no microcracks were observed in the T1_end thin section, their presence cannot be entirely excluded. Phyllosilicate-rich rocks may swell significantly through moisture absorption, generating interfacial pressures that can induce concrete cracking [38].

Both coarse and fine aggregates demonstrate alkali–silica reaction (ASR) potential due to the presence of polycrystalline and monocrystalline quartz with undulose extinction [39,40]. Microscopic analysis revealed gel formation in cement paste and aggregates, surrounding some aggregates and filling fractures (Figure 9a,e and Figure 10a,d,e). Specimens contained voids that were either unfilled or partially filled with gel and cryptocrystalline material. This cryptocrystalline material likely results from the precipitation of soluble salts and/or sulfates detected by spectrophotometry (Table 3).

As described by Pole and Sims, cryptocrystalline reaction products may originate from precursor gel formed through internal aggregate reactions [41]. This precursor gel transforms into clear amorphous gel upon contact with the more alkaline cement paste. Microfractures in undulose and elongated quartz grains may provide migration pathways for alkali solutions within the cement paste, facilitating reactions at favorable sites [38].

All concrete tie samples exhibited ASR processes and products of varying intensity. Carbonation was observed in both concrete types, with T1_end showing advanced cement paste and fine aggregate carbonation. T1_central displayed carbonation restricted to coarse aggregates, while T2_end exhibited only incipient carbonation.

### 3.5. XRD/SEM-EDS Analysis

**Figure 11:**
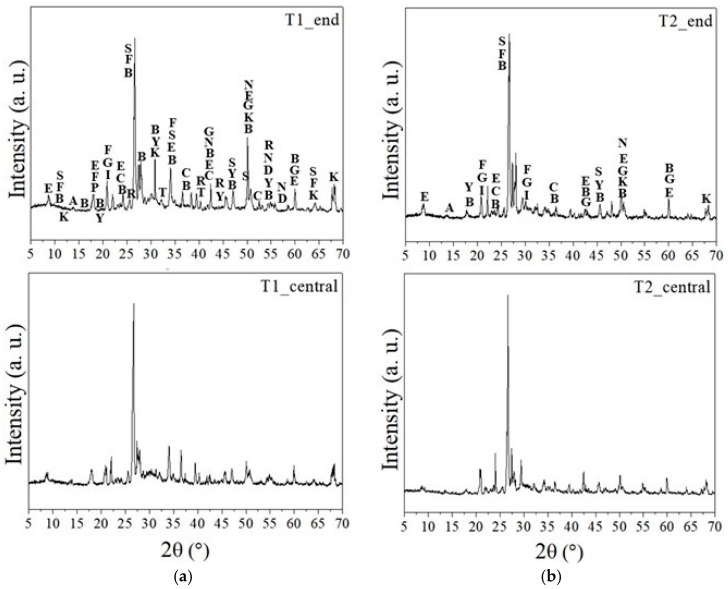
XRD patterns of the main phases identified in the samples of concrete ties: (**a**) T1; (**b**) T2. Subtitle: abbreviation—compound name/mineral. S—calcium silicate hydrate/C-S-H; F—calcium aluminum silicate hydrate/C-A-S-H; P—portlandite; R—natron; Y—ye’elimite; A—AFm phases; E—ettringite; G—gypsum; C—calcite; D—dolomite; B—boggsite; I—misenite; K—arcanite; N—aluminum hydroxide.

Figure 11 shows the diffraction patterns of samples taken from the end and central region of the concrete ties (T1 and T2). The presence of typical cement hydration compounds was observed, including portlandite, calcium silicate hydrate (C-S-H), calcium aluminum silicate hydrates (C-A-S-H), and various phases of sulphoaluminate hydrates (AFm—monosulphoaluminate calcium hydrate, ettringite, gypsum), as well as non-hydrated phases like ye’elimite, calcite, and dolomite. Aluminum sulfates and aluminum hydrate were also identified. All phases identified by X-ray diffraction are detailed in Table A1 in Appendix A.

**Figure 12:**
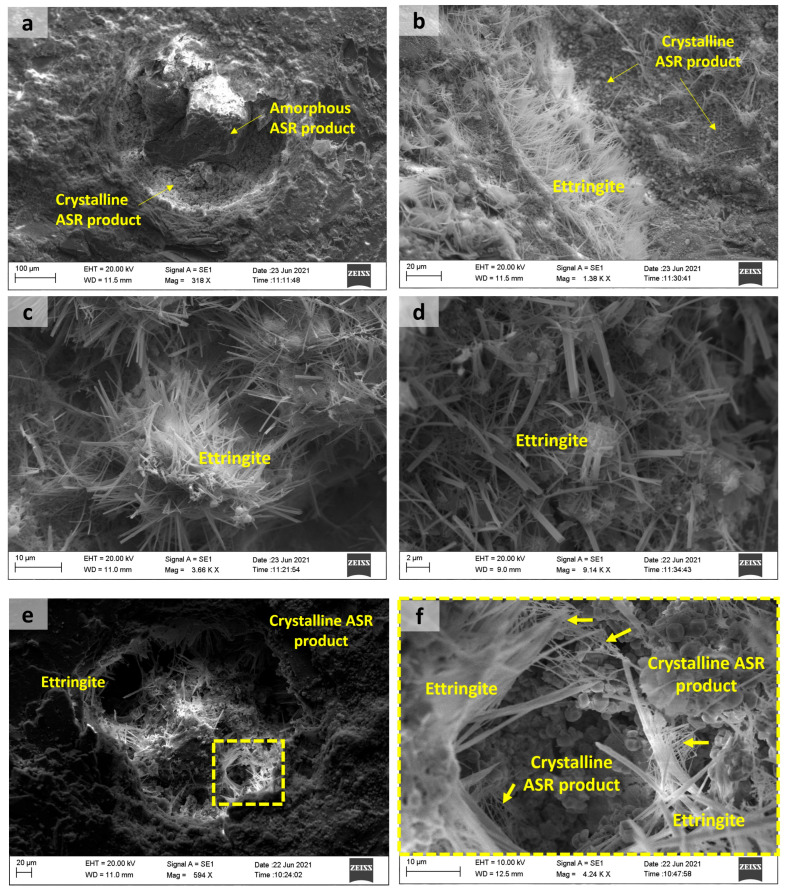
Pores filled with amorphous and crystalline ASR products (**a**) T1_end; (**b**) T2_central; ettringite and crystalline products on pores (**c**) T1_end; (**d**) T2_central; ettringite on the cement paste: (**e**) T2_end; (**f**) T2_central.

Ettringite was observed in T1_end, T1_central, and T2_central samples. Crystals of ettringite were detected by SEM analysis in air voids and on the surface of aggregate and cement paste, as shown in Figure 12. Ettringite within air voids or cracks is unlikely to directly contribute to concrete damage, as it forms in relatively large spaces without causing cracking or disruption to the concrete ties. However, the presence of this phase was also noted in the cement paste. In this case, secondary ettringite formation can contribute to the expansion mechanism and cracking of the concrete. Awashi et al. also observed the deposition of delayed ettringite at aggregate interfaces, inside cracks, and in the cement paste of concrete ties in Indian railways after 6–9 years of environmental exposure [8].

**Figure 13:**
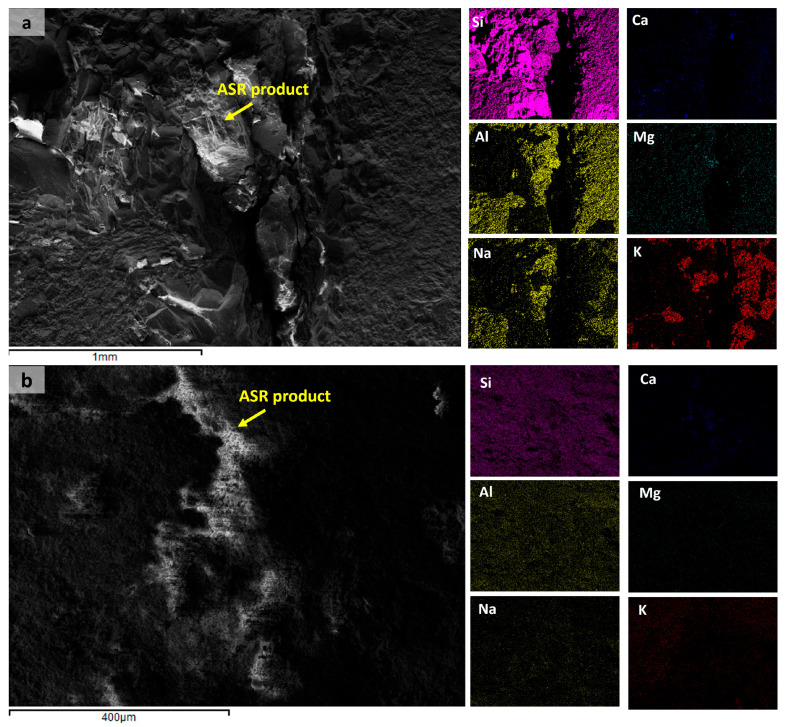
Mapping of chemical elements present in microcracks (**a**) between aggregates in a sample taken from T1; (**b**) aggregates and cement paste in a sample from T2 concrete tie.

In addition to calcium and magnesium carbonates, the results revealed carbonation products from sodium, such as thermonatrite and natron. The T2_central samples did not show either of these sodium carbonate phases, while natron was observed only in the T1 samples. All samples exhibited the presence of boggsite phase, indicating the formation of (N,C)ASH gels. The XRD results also identified arcanite, misenite, and goergeyite compounds, which contain potassium in their composition. The presence of potassium and/or sodium, along with a limited amount of calcium, is essential to form ASR products [42]. The presence of portlandite favors ASR because it provides Ca^2+^ ions in solution. Small amounts of available alkalis (0.76%) do not lead to the formation of ASR products, resulting in the occurrence of non-deleterious expansion [43]. Figure 13 shows the presence of these elements in microcracks between aggregates and/or cement paste in samples taken from T1 and T2 concrete ties.

**Figure 14:**
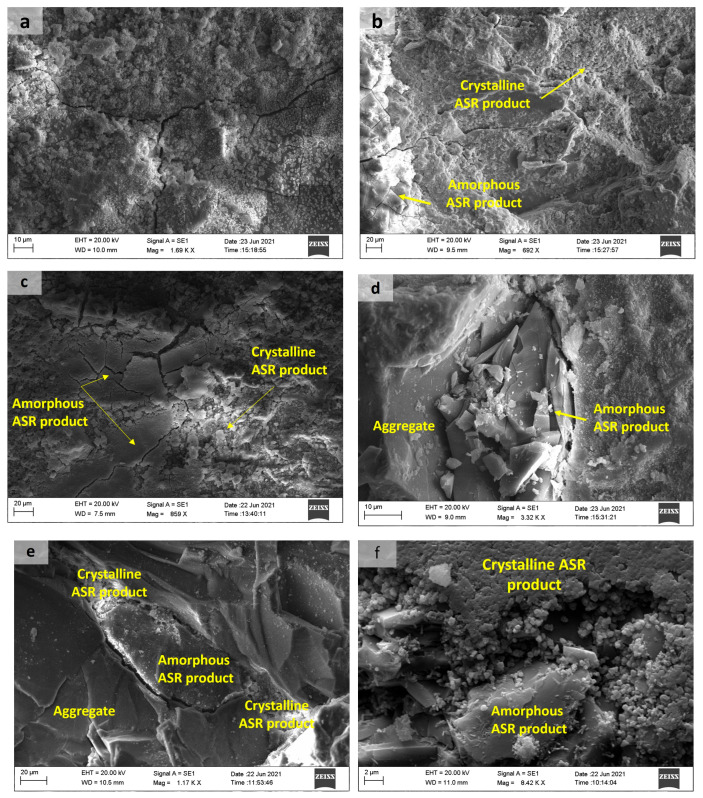
(**a**) Microcracks and ASR products on the aggregate surface; amorphous and crystalline products in cracked aggregate and microcracks present in concrete ties: (**b**) T1_central; (**c**) T2_end; (**d**) T1_central; and (**e**,**f**) T2_central.

**Figure 15:**
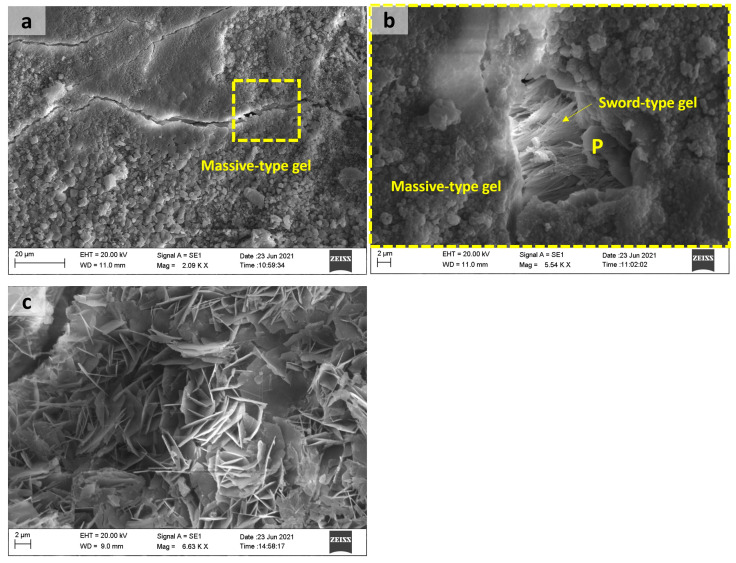
T1_end sample: (**a**) massive-type gel on the surface of aggregate; (**b**) sword-type gel present in microcracks; T1_central: (**c**) rosette-type reaction products in pores. Note: P—portlandite.

ASR products were identified at aggregate–paste interfaces, within aggregates, and in pores/microcracks of concrete ties, as show in Figure 14 and Figure 15. All samples exhibited characteristic distributions of both amorphous and crystalline phases, as exemplified in Figure 14. The primary distinction between these ASR products lies in their Na/K ratios, with amorphous phases exhibiting higher values [44]. Initially, amorphous ASR products form, inducing aggregate cracking. Subsequently, crystalline ASR products develop, filling the open cracks. Notably, crystalline products may also precipitate independently during later ASR stages, bypassing amorphous precursors. This indicates that their formation correlates with reaction progression rather than deriving from amorphous-phase transformation [44]. The complete sequence of ASR-induced concrete deterioration is extensively detailed in [45].

Figure 15 shows sodium and calcium silicate reaction products with massive and sword-type gel morphology formed in the T1_end sample, and a rosette-type reaction product was observed in the pores of the aggregates in T1_central samples. The presence of portlandite crystals was also observed. Crystals with a sword-type morphology and a rosette-type product, corresponding to a sodium–calcium silicate hydrate, can be associated with a more advanced stage of ASR [19]. Shi et al., through the synthesis of ASR products similar to rosette-type ones, verified that temperature affects the structural formation of this crystalline ASR product. They also suggested that the swelling of a layered silicate-sheet structure by water uptake is not the mechanism of ASR-induced expansion at temperatures between 40 and 80 °C [46].

The deterioration of concrete ties observed in this study resulted from a complex interaction of chemical, mechanical, and environmental factors that synergistically accelerate structural degradation. Alkali–silica reaction (ASR) produces expansive gels while sulfate attack leads to the formation of ettringite and gypsum crystals, creating internal tensile stresses that can initiate microcracking within the concrete matrix. The damage begins at the microscopic level but progressively weakens the overall structure.

The prestressing force in concrete ties creates a non-uniform stress field that significantly influences degradation patterns. As the prestressing force diminishes toward the tie ends, these regions become particularly vulnerable to damage. The combination of reduced prestress and existing internal stresses from swelling reactions leads to stress concentrations that accelerate crack formation and propagation. This mechanical aspect of deterioration explains why damage is more severe at the tie ends.

Environmental conditions play a crucial role in accelerating the deterioration process. Capillary action serves as the primary mechanism for transporting water and aggressive substances into the concrete pore structure. The presence of moisture not only facilitates chemical reactions but also affects their rates and progression [47]. The critical role of water exposure is evidenced by significantly greater damage in poorly drained ties (T1) compared to their well-drained counterparts (T2), as shown in Figure 1, demonstrating how environmental factors can outweigh mechanical properties in determining service life.

Water availability governs reaction dynamics: continuous exposure promotes rapid ASR and delayed ettringite formation [21], while wet–dry cycles influence sulfate ion transport and crystallization patterns [48]. A distinct saturation threshold exists, where combined saturated and cyclic conditions maximize pore structure damage [48]. There is a critical point between saturated sulfate attack and drying–wetting cycles that intensifies the damage to the internal structure of concrete pores [49].

These mechanisms establish a degradation cycle: initial chemical reactions weaken the concrete matrix, increasing susceptibility to stress-induced cracking. This crack, in turn, creates pathways for the deeper penetration of moisture and aggressive ions, further accelerating chemical degradation. This feedback loop explains the nonlinear progression of deterioration, where damage rates escalate over time despite consistent environmental exposure. These findings highlight the need for comprehensive durability assessments that consider these interconnected factors in railway infrastructure design and maintenance.

### 3.6. A Predictive Model of ASR Damage: Integrating Expansion Kinetics and Transport Properties

Empirical models, such as the Larive model [50], are widely used to simulate the expansion kinetics of alkali–silica reaction (ASR) in concrete. The model describes ASR-induced expansion as a sigmoidal function of time, characterized by three empirical parameters: the asymptotic expansion (*ε*_∞_)—ultimate strain under unrestrained conditions; the latency period (*τ*_*lat*_)—initial phase with minimal expansion; and the characteristic time (*τ*_*k*_)—controls the rate of expansion during the rapid-growth phase. The model captures the typical three-phase behavior observed in ASR progression: an initial latency period with no visible expansion, a rapid expansion phase, and a final stabilization phase.

Building on Larive’s formulation, Multon and Toutlemonde [51] developed a chemo-mechanical framework that accounts for stress effects on ASR. Their model treats ASR-induced swelling as a volumetric strain which redistributes under mechanical restraint (e.g., prestressing or confinement). Crucially, while total volumetric expansion remains nearly constant, mechanical restraints cause anisotropic expansion: greater deformation in less-confined directions and suppressed expansion along highly stressed axes.

This “expansion transfer” mechanism is clearly observed in prestressed concrete ties, where stress gradients lead to non-uniform ASR damage. Table 5 summarizes the strength degradation in ties T1 and T2 after 10 years.

The data clearly demonstrate that prestress significantly inhibits internal swelling degradation. In central regions, where compressive stresses are highest, concrete retains close to its original strength (e.g., T2_central: 64 MPa to 66 MPa after 10 years). Conversely, anisotropic damage patterns emerge in less confined areas. End regions—subject to lower prestress—exhibit severe strength loss (e.g., T1_end: 66% reduction). This aligns with Multon and Toutlemonde’s expansion transfer theory: mechanical restraint redistributes ASR swelling, concentrating damage in directions of minimal confinement. The result is a stark strength gradient along the tie’s length, underscoring how stress states govern ASR’s spatial progression.

Beyond mechanical effects, ASR profoundly alters transport properties. Maalouf et al. [52] showed that internal swelling reactions alter concrete’s microstructure, increasing air permeability by up to 44 times and water diffusivity by 3x at just 0.22% expansion. These microstructural changes create pathways for aggressive agents, accelerating the deterioration process. Our experimental data on prestressed concrete ties reveal even more extreme degradation in the field conditions: severely affected T1_end regions exhibiting 10x capillary absorption coefficients and 47x chloride migration coefficients greater than the central zones. This spatial variation directly correlates with damage severity, where low-prestressed regions develop extensive microcrack networks while high-stress areas retain lower permeability. Particularly concerning T1_end, this not only confirms Maalouf et al.’s findings but demonstrates even more extreme transport degradation in real-world conditions. This synergy between expansion, cracking, and permeability creates a self-accelerating cycle, where initial ASR damage enables the faster penetration of harmful substances, which in turn exacerbates the damage. These findings underscore that comprehensive durability assessments must account for both mechanical property degradation and transport property evolution, as they collectively govern service life through combined mechanical and chemical attack mechanisms.

Therefore, future predictive tools should ideally incorporate not only the time-dependent expansion behavior modeled from empirical data but also the interaction with stress states and the concurrent evolution of transport properties. Such a model would enhance condition assessments and service life predictions, particularly for critical elements like prestressed railroad ties, where stress gradients and environmental exposure drive complex degradation. 

### 3.7. Practical Recommendations and Future Research

Based on our findings, we recommend four key strategies to enhance prestressed concrete tie durability:

(1) Material optimization: Use non-reactive aggregates with low-alkali cement and supplementary cementitious materials to mitigate ASR/DEF risks by controlling reactive species availability;
(2) Design improvements: Implement optimized prestress distribution focusing on vulnerable end regions to improve stress resistance;
(3) Drainage system: Install properly graded drainage systems to minimize water retention and associated deterioration mechanisms;
(4) Monitoring protocol: Establish regular visual inspections and non-destructive testing (NDT) programs, prioritizing examination of rail seats and tie ends for early deterioration detection.

These recommendations are supported by our observations that severe deterioration occurred in poorly drained sections and that end regions exhibited higher crack density than central zones. 

For future research, priority should be given to the implementation of integrated monitoring systems combining long-term field observation with accelerated laboratory testing to improve durability assessment frameworks; development of robust predictive models that account for material properties, microenvironmental exposure, and traffic loading patterns; and exploration of advanced material technologies including advanced composite materials.

## 4. Conclusions

This study employed a comprehensive set of analytical techniques to investigate deterioration mechanisms in two groups of prestressed concrete ties. Despite being manufactured by the same producer under similar conditions and using comparable materials, the two groups exhibited varying levels of degradation. The key findings include the following:

- Both groups showed a reduction in compressive strength compared to the 28-day benchmark. Specimens taken from the tie ends exhibited lower strength than those from the center. T1 samples showed a strength reduction of up to 66%, while T2 samples decreased by approximately 40%.
- T1 samples exhibited significantly higher capillary absorption and chloride migration coefficients (D_nss_) than T2, indicating increased permeability. This facilitated the ingress of aggressive agents, leading to higher concentrations of soluble salts and an accelerated deterioration process.
- Petrographic analysis revealed that both coarse and fine aggregates in T1 and T2 were found to be potentially reactive. Microcracking and voids filled with gel and cryptocrystalline materials were observed. T1 samples from the ends showed advanced carbonation, while central samples exhibited carbonation primarily around aggregate boundaries.
- XRD analysis identified ettringite in T1 ends and T2 centers, with SEM images showing ettringite in air voids and on aggregate surfaces. This phase in the cement paste is linked to secondary ettringite formation, contributing to expansion and cracking.
- ASR products were identified both at aggregate–paste interfaces and within aggregates, pores, and microcracks. All specimens contained boggsite, characteristic of (N,C)ASH gel development. Furthermore, T1 displayed thermonatrite and natron, which are typical carbonation products of sodium. Massive crystals with a sword-type morphology and rosette-type reaction products associated with a more advanced stage of ASR were found in T1 samples.
- The level of deterioration in T1 ties is more severe than in T2, likely due to increased moisture exposure and drying cycles. The superior performance of T2 ties underscores the critical role of drainage design in durability.
- The key determinant of performance variation between T1 and T2 ties is their differing microenvironmental moisture exposure, interacting critically with prestress distribution effects. The synergistic combination of (1) terrain-induced moisture accumulation disparities and (2) stress confinement patterns create the significant durability gap observed in field performance.

This study demonstrates the critical interplay between internal swelling reactions, moisture exposure, and mechanical performance in concrete ties. Our findings reveal that the combination of ASR-induced damage and water absorption significantly compromises tie durability, particularly in low-prestress zones with poor drainage. To address these challenges, we highlight two key approaches: (1) developing advanced predictive models that integrate ASR kinetics, stress redistribution effects, and transport property evolution; (2) implementing practical engineering solutions including optimized prestress distribution designs and targeted drainage maintenance in vulnerable areas. These mitigation strategies offer substantial potential to extend service life, reduce maintenance costs, and enhance the sustainability of railway infrastructure by addressing the root causes of premature deterioration.

## Figures and Tables

**Table 1:** Characteristics of concrete used for production of ties.

Characteristics of Concrete Ties		T1	T2
Slump (cm)		4.21 ± 1.24	3.66 ± 1.50
Variation in time of curing (h)		5 to 9	5:30 to 15
Average time of curing (h)		6.45	6.44
Compressive strength ^1^ (MPa) at	3 days	45.3 ± 3.9	50.5 ± 4.2
	7 days	52.2 ± 4.6	57.0 ± 3.9
	28 days	58.9 ± 4.8	64.0 ± 4.2
3 point-flexural tensile strength ^2^ (MPa) at 28 days		7.9 ± 0.9	6.3 ± 0.5

^1^ Average and standard deviation of cylindrical specimens prepared in 100/200 mm dimensions. ^2^ Average and standard deviation of 150 × 150 mm specimens (40 mm support spacing).

**Table 2:** Spatial distribution of mechanical properties of concrete ties (T1 and T2) after 10 years in service.

	T1_End	T1_Central	T2_End	T2_Central
Compressive strength (MPa)	20.0 ± 8.1	30.0 ± 1.9	38.5 ± 8.9	66.0 ± 0.2
Dynamic elastic modulus (GPa)	28.8 ± 5.4	40.8 ± 4.4	32.9 ± 8.6	47.0 ± 4.8

**Table 3:** Spatial distribution of soluble salts, sulfates, and chlorides in concrete ties.

	T1_End	T1_Central	T2_End	T2_Central
Soluble salt (%)	2.17 ± 0.01	2.19 ± 0.01	1.26 ± 0.07	0.96 ± 0.02
Sulfates (%)	0.10 ± 0.00	0.10 ± 0.00	0.11 ± 0.01	0.05 ± 0.01
Chloride (%)	<0.0062	<0.0062	<0.0062	<0.0062

**Table 4:** Petrographic characteristics of studied concrete ties.

	Parameter	T1_End	T1_Central	T2_End	T2_Central
Coarse aggregate	Rock type	granite	granite	granitoid	granite
	Particle size	5–25 mm	5–25 mm	5–25 mm	5–25 mm
	Microcracks	punctual	occasional	occasional	occasional
	Reactivity	potentially	potentially	potentially	potentially
	Reaction edges	incipient	incipient	incipient	incipient
	Gel	thin film	thin film	thin film	thin film
Fine aggregate	Mineralogy	quartz	quartz	quartz	quartz
	Microcracks	no detected	no detected	no detected	no detected
	Reaction edges	sinous	sinous	sinous	sinous
	Gel	present	present	present	present
Cement paste	Color	uniform	uniform	uniform	uniform
	Voids	partially filled	partially filled	partially filled	partially filled
	Gel	present	present	present	present
	Carbonation	advanced	restricted	no detected	no detected
Concrete	ASR	present	present	present	present

**Table 5:** ASR severity gradients in prestressed ties (T1 and T2) after 10 years in service.

Region	Initial f_c_ (MPa)	10-Year f_c_ (MPa)	Strength Loss (%)	ASR Severity
T1_end	58.9	20.0 ± 8.1	~66%	Severe (low prestress)
T1_central	58.9	30.0 ± 1.9	~49%	Moderate (partial σ inhibition)
T2_end	64.0	38.5 ± 8.9	~40%	Moderate
T2_central	64.0	66.0 ± 0.2	~0%	Negligible (high σ)

## Data Availability

The original contributions presented in this study are included in the article. Further inquiries can be directed to the corresponding author.

## Abbreviations

The following abbreviations are used in this manuscript:

ASR	Alkali–Silica Reaction
CAGR	Compound Annual Growth Rate
DEF	Delay Ettringite Formation
EDS	Energy Dispersive Spectroscopy
RSD	Rail-Seat Deterioration
SEM	Scanning Electron Microscopy
XRD	X-Ray Diffraction (XRD)

## Appendix A

**Table A1:** Phases identified by X-ray diffraction in at least one sample taken from the end and central positions of concrete ties studied. Note: P—present; n.d—not detected.

Compounds	Chemical Formula	T1		T2	
		End	Central	End	Central
Quartz	$SiO_{2}$	P	P	P	P
C-S-H	-	P	P	P	P
C-A-S-H	-	P	P	P	P
Portlandite	$Ca({OH)}_{2}$	P	P	P	P
AFm	${Ca}_{4}{Al}_{2}SO_{10}{\cdot 16H}_{2}O$	P	P	P	P
Ettringite	${Ca}_{6}{Al}_{2}{\left(SO_{4}\right)}_{3}{\left(OH\right)}_{12}\cdot 26H_{2}O$	P	P	P	P
Gypsum	$CaSO_{4}\cdot 2H_{2}O$	P	P	P	P
Calcite	$CaCO_{3}$	P	P	P	P
Dolomite	$CaMg{\left(CO_{3}\right)}_{2}$	P	P	P	P
Thermonadrite	${Na}_{2}CO_{3}\cdot H_{2}O$	P	P	P	n.d
Natron	${Na}_{2}CO_{3}\cdot 10H_{2}O$	P	P	n.d	n.d
Boggsite	${Na}_{3.7}{Ca}_{7}4{Al}_{18.5}{Si}_{77.5}O_{192}7{4H}_{2}O$	P	P	P	P
Aluminum sulfate	${Al}_{2}{\left({SO}_{4}\right)}_{3}$	P	P	P	P
Nordstrandite	$Al{\left(OH\right)}_{3}$	P	P	P	P
Ye’elimite	${Ca}_{4}{Al}_{6}O_{12}{(SO}_{4})$	P	P	P	P
Arcanite	$K_{2}{SO}_{4}$	P	P	P	P
Misenite	$K_{8}{SO}_{4}{\left(SO_{3}OH\right)}_{6}$	P	P	P	P
Goergeyite	$K_{2}{Ca}_{5}{\left(SO_{4}\right)}_{6}H_{2}O$	P	P	P	P

Reference code: Calcium silicate hydrate (C-S-H—29–0377; 033–0306; 042–0538); calcium aluminum silicate hydrate (C-A-S-H—046–1405; 20–0452); calcium hydroxide (portlandite—Ca(OH)_2_—44–1481); calcium aluminum sulfate (ye’elimite—Ca_4_Al_6_SO_16_—033–0256); calcium aluminum sulfate hydrate (AFm phases—Ca_4_Al_2_SO_10_·16H_2_O—044–0602); ettringite (Ca_6_Al_2_(SO_4_)_3_(OH)_12_·26H_2_O—41–1451); calcium sulfate dihydrate (gypsum—CaSO_4_·2H_2_O—021–0816); calcium carbonate (calcite—CaCO_3_—05–0586); calcium magnesium carbonate (dolomite—CaCO_3_·MgCO_3_—036–0426); sodium calcium aluminum silicate hydrate—(boggsite—Na_3.7_Ca_7.4_Al_18.5_Si_77.5_O_192_74H_2_O—042–1379); potassium sulfate hydroxide (misenite—K_8_(SO)_4_(SO_3_OH)_6_—41–1363); potassium sulfate (arcanite—K_2_SO_4_—05–0613); aluminum sulfate (millosevichite—Al_2_(SO_4_)_3_—42–1410); aluminum hydroxide (nordstrandite—Al_2_(OH)_3_—24–0006).
